# Short Treatment of 42 Days with Oral GS-441524 Results in Equal Efficacy as the Recommended 84-Day Treatment in Cats Suffering from Feline Infectious Peritonitis with Effusion—A Prospective Randomized Controlled Study

**DOI:** 10.3390/v16071144

**Published:** 2024-07-16

**Authors:** Anna-M. Zuzzi-Krebitz, Katharina Buchta, Michèle Bergmann, Daniela Krentz, Katharina Zwicklbauer, Roswitha Dorsch, Gerhard Wess, Andrea Fischer, Kaspar Matiasek, Anne Hönl, Sonja Fiedler, Laura Kolberg, Regina Hofmann-Lehmann, Marina L. Meli, Andrea M. Spiri, A. Katrin Helfer-Hungerbuehler, Sandra Felten, Yury Zablotski, Martin Alberer, Ulrich von Both, Katrin Hartmann

**Affiliations:** 1 LMU Small Animal Clinic, Centre for Clinical Veterinary Medicine, LMU Munich, 80539 Munich, Germany; k.buchta@medizinische-kleintierklinik.de (K.B.); michele.bergmann@lmu.de (M.B.); d.krentz@medizinische-kleintierklinik.de (D.K.); k.zwicklbauer@medizinische-kleintierklinik.de (K.Z.); roswitha.dorsch@lmu.de (R.D.); gwess@lmu.de (G.W.); andreafischer@lmu.de (A.F.); a.hoenl@medizinische-kleintierklinik.de (A.H.); y.zablotski@med.vetmed.uni-muenchen.de (Y.Z.); hartmann@lmu.de (K.H.); 2 Institute of Veterinary Pathology, Centre for Clinical Veterinary Medicine, LMU Munich, 80539 Munich, Germany; kaspar.matiasek@neuropathologie.de (K.M.); sonja.fiedler@patho.vetmed.uni-muenchen.de (S.F.); 3 Division of Paediatric Infectious Diseases, Dr. von Hauner Children’s Hospital, University Hospital, LMU Munich, 80337 Munich, Germany; laura.kolberg@med.uni-muenchen.de (L.K.); martin.alberer@lrz.uni-muenchen.de (M.A.); ulrich.von.both@med.uni-muenchen.de (U.v.B.); 4 Clinical Laboratory, Department of Clinical Diagnostics and Services, and Center for Clinical Studies, Vetsuisse Faculty, University of Zurich, CH-8057 Zurich, Switzerland; regina.hofmann-lehmann@uzh.ch (R.H.-L.); mmeli@vetclinics.uzh.ch (M.L.M.); aspiri@vetclinics.uzh.ch (A.M.S.); khungerbuehler@vetclinics.uzh.ch (A.K.H.-H.); 5 Clinic for Small Animal Internal Medicine, Vetsuisse Faculty, University of Zurich, CH-8057 Zurich, Switzerland; sandra.felten@uzh.ch; 6 German Center for Infection Research (DZIF), Partner Site Munich, 80337 Munich, Germany

**Keywords:** FIP, feline coronavirus, FCoV, treatment duration, therapy, antiviral chemotherapy, GS-441524, BOVA

## Abstract

In the past, feline infectious peritonitis (FIP) caused by feline coronavirus (FCoV) was considered fatal. Today, highly efficient drugs, such as GS-441524, can lead to complete remission. The currently recommended treatment duration in the veterinary literature is 84 days. This prospective randomized controlled treatment study aimed to evaluate whether a shorter treatment duration of 42 days with oral GS-441524 obtained from a licensed pharmacy is equally effective compared to the 84-day regimen. Forty cats with FIP with effusion were prospectively included and randomized to receive 15 mg/kg of GS-441524 orally every 24h (q24h), for either 42 or 84 days. Cats were followed for 168 days after treatment initiation. With the exception of two cats that died during the treatment, 38 cats (19 in short, 19 in long treatment group) recovered with rapid improvement of clinical and laboratory parameters as well as a remarkable reduction in viral loads in blood and effusion. Orally administered GS-441524 given as a short treatment was highly effective in curing FIP without causing serious adverse effects. All cats that completed the short treatment course successfully were still in complete remission on day 168. Therefore, a shorter treatment duration of 42 days GS-441524 15 mg/kg can be considered equally effective.

## 1. Introduction

Feline infectious peritonitis (FIP) is a highly fatal disease of domestic cats caused by feline coronavirus (FCoV) [1]. However, in the last few years, experimental and field studies have demonstrated that treatment with effective drugs, such as oral and injectable GS-441524 [2,3,4,5,6,7,8,9], oral and injectable remdesivir (GS-5734) [10,11,12,13], or injectable 3C-like protease inhibitor GC376 [14,15], as single drugs or in various combinations can lead to remission of FIP. The antiviral nucleoside analogue GS-441524 is currently the most promising treatment option, showing excellent results in terms of efficacy and survival rate [2,3,4,5,6,7,8,9,10,11,12,13,14,15,16,17]. In one experimental study, 10/12 specific pathogen-free cats, experimentally infected with FIP-causing FCoV and showing clinical signs of FIP, were treated with GS-441524 at a dosage of 2–5 mg/kg applied subcutaneously (SC) every 24h (q24h) for 14 days. All cats were in remission at the time of publication [2]. One study evaluated the efficacy of GS-441524 (2–4 mg/kg, SC, q24h) in 31 cats with naturally acquired FIP over 84 days. In this study, 24/31 cats were in sustained remission at the time of publication [3]. In a case series, four cats with naturally acquired FIP with central nervous system (CNS) involvement were treated with GS-441524 (5–10 mg/kg, SC, q24h) for at least 12 weeks. At the time of publication, three of the four cats were alive without clinical signs and one cat was euthanized after 216 days (following two courses of treatment with GS-441524) [5]. One study investigated the efficacy of GS-441524 in combination with the 3C-like protease inhibitor GC376, both administered subcutaneously in different dosages, with 45/46 cats surviving. Due to severe progression of the disease, one cat was euthanized on day 2 of the treatment [15]. The first published prospective study of the authors’ research group to evaluate the efficacy of GS-441524 applied as an oral multicomponent drug showed excellent efficacy. The compound used in this study was produced under uncontrolled conditions in China by Mutian Life Sciences Limited. In addition to GS-441524, the compound contained herbal preparations. Eighteen cats diagnosed with naturally acquired FIP were included; cats without neurologic and/or ocular signs received 5 mg/kg of the active compound, while cats with neurologic and/or ocular signs received 10 mg/kg (when relying on the manufacturer’s information) [8]. However, an analysis of the multicomponent drug by liquid chromatography and mass spectrometry (performed after the publication of the referenced study) revealed that the tablets contained more than double the amount of GS-441524 than stated by the manufacturer (personal communication J. Horak). All 18 cats completed the treatment of 84 days successfully and were clinically healthy [8]. Also, viral RNA loads decreased in blood, effusions, and feces during the oral treatment with GS-441524 [18]. A long-term follow-up study of these 18 cats proved that treatment with GS-441524 was effective against FIP in both the short and long term [17]. All cats were still alive more than one year after the diagnosis (except one cat that died in a car accident). In all but one cat, FCoV RNA could not be detected in blood during the follow-up period. This cat tested FCoV RNA-positive in blood before treatment and became negative from day 7 onwards, but at the recheck at week 24 tested positive with a very low viral load but without clinical signs. On all subsequent rechecks, this cat tested FCoV RNA-negative. No cat experienced a relapse [17]. The authors’ research group also published a report on a clinical follow-up and postmortem findings of a cat cured from FIP treated with the GS-441524-containing multicomponent compound that died in a car accident. Neither signs of FIP, nor FCoV RNA or antigen could be detected in postmortem examinations by quantitative reverse transcription polymerase chain reaction (RT-qPCR) and immunohistochemistry (IHC) in any tissues or body fluids, including the feces of this cat [16].

In summary, the previous studies of the authors’ research group demonstrated that cats with FIP showed rapid clinical and laboratory improvement when treated with an oral compound containing GS-441524. By day 14, not only had clinical signs disappeared, but also viral RNA was no longer detectable in the blood of the cats, indicating that all cats had cleared viremia [8]. Along the same lines, a study including 60 client-owned domestic cats with naturally occurring FIP demonstrated that viral nucleic acids became undetectable in ascitic fluid or abdominal lymph node tissue by day 11 post-treatment initiation [19]. Accordingly, it was questioned whether the duration of treatment, which is currently recommended to be 84 days, could be shortened. A longer treatment duration is associated with higher costs, a potentially increased risk of side effects, and logistical and operational stress for the cats and their owners. Many cat owners struggle with expensive treatment costs and might thus rather choose to euthanize their cats than opt for treatment despite the good prognosis when treated with GS-441524. Therefore, the aim of the present study was to evaluate whether a shorter treatment duration of 42 days (six weeks) with GS-441524 would be as effective as the longer, currently recommended treatment duration of 84 days (12 weeks) in a prospective randomized controlled study. 

## 2. Materials and Methods

### 2.1. Study Design

The present study received approval from the Government of Upper Bavaria (reference number 55.2-2532.Vet_02-20-52) and obtained ethical approval from the Institutional Review Board of the Centre for Clinical Veterinary Medicine at LMU Munich (reference number 261-19-03-2021). The research adhered to the established German guidelines for prospective studies. Owners had to give their written consent to participate.

**Figure 1:**
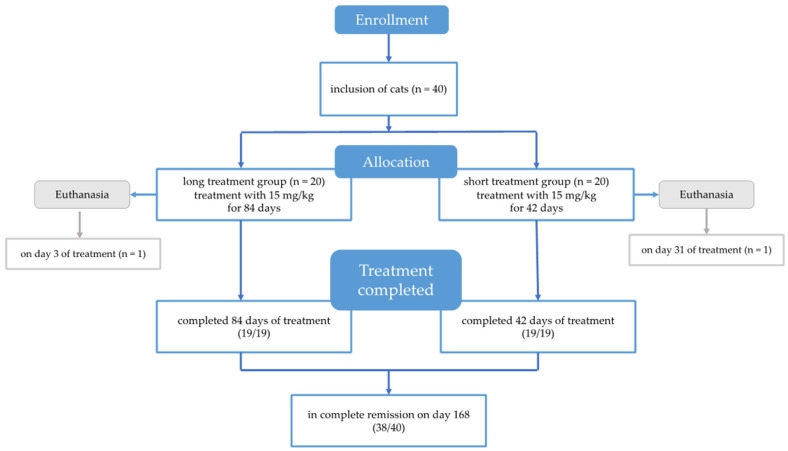
Flow diagram illustrating enrollment, inclusion, process of randomized allocation to long and short treatment group, and outcome of cats in the study.

The study design was a prospective randomized controlled treatment trial. A total of 40 cats with FIP were prospectively included (Figure 1). Cats were randomly assigned to one of two treatment arms using envelopes as an allocation concealment method (www.random.org (accessed on 5 September 2022)). Allocation to groups was blinded until day 7 of the study, when envelopes were opened. Cats in group 1 (20 cats) received treatment with GS-441524 for 84 days (long treatment group), and cats in group 2 (20 cats) received treatment with GS-441524 for a shorter treatment duration of 42 days (short treatment group). All cats were treated in-house for the first seven days and then monitored with regular visits to the hospital for the assessment of clinical and laboratory parameters and viral loads following a predetermined schedule up to 84 days (Table 1). The primary outcome parameter was the remission rate at day 84. Cats were then presented again on day 168 for confirmation of their health status. Secondary outcome parameters were changes in clinical and laboratory parameters and viral loads. 

All 40 cats were administered the same dosage of 15 mg/kg, per os (PO), q24h of GS-441524. The number of tablets given was adjusted to the current weight. Cats received tablets daily at the same time following a one-hour fasting period. Half an hour after tablet administration, food was provided to the cats. During the first seven days of treatment (day 1 to day 7) cats were monitored closely in the hospital under medical care by board-certified specialists in internal medicine and intensive care 24h per day. Supportive measures, symptomatic treatment, as well as diagnostic procedures were applied as necessary. The costs for the GS-441524 tablets were paid by the owners. 

**Figure 2:**
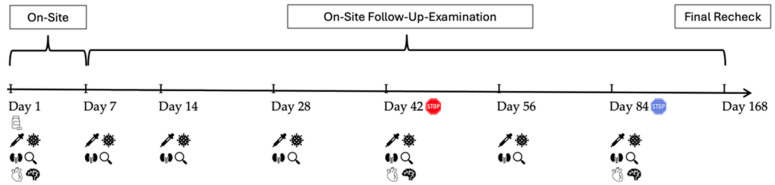
Timeline visualizing the in-hospital and on-site follow-up examinations throughout the study course. 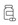: start of treatment with GS-441524; 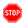: end of treatment of 20/40 cats on day 42; 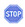: end of treatment of 20/40 cats on day 84; 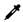: evaluation of laboratory parameters; 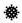: measurement of virology parameters (including viral loads in blood, effusion and feces, anti-FCoV antibodies); 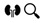: abdominal ultrasonography; 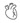: cardiologic examination; 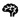: neurologic examination.

On-site follow-up examinations were performed on days 14, 28, 42, 56, and 84 after the start of treatment. In addition, cats were presented again 168 days after the start of treatment for a final recheck. (Figure 2). Complete remission was defined as (1) a body condition score of ≥4/9 [20], (2) normothermia, (3) the absence of any clinical signs of FIP [21,22], (4) a modified Karnofsky’s score of at least 90%, and (5) the normalization of laboratory parameters typically altered in FIP cats (non-regenerative anemia, microcytosis, lymphopenia, thrombocytopenia, neutrophilia, hyperglobulinemia, hypoalbuminemia, hyperbilirubinemia, low albumin/globulin ratio, increase in serum amyloid A (SAA), and alpha-1-acid-glycoprotein (AGP)) [21,22,23]. Cats that died during the study course underwent a full postmortem examination, including necropsy, histology, and immunohistochemical examination for FCoV antigen [16].

### 2.2. Compound

The compound GS-441524 was provided by BOVA Specials, London, UK, as tablets containing 50 mg of GS-441524 each. In addition to GS-441524, the tablets contained citric acid, magnesium stearate, microcrystalline cellulose, mannitol, sodium starch, silicon dioxide, and tuna flavor. GS-441524 is not licensed but is legally manufactured and distributed by BOVA Specials, London, UK, in a strictly controlled manner. Due to a special import permit released from the Government of Upper Bavaria specifically for this study, BOVA GS-441524 tablets were legally imported for this study. 

### 2.3. Inclusion Criteria and Study Cohort

Client-owned cats were recruited for the study (Table 2 and Table S1). Inclusion criteria were (1) diagnosis of FIP (as defined below), (2) presence of abdominal and/or pleural effusion, (3) body weight of at least 2 kg, (4) negative test results for feline immunodeficiency virus (FIV) antibodies and feline leukemia virus (FeLV) antigen, and (5) absence of other severe diseases unrelated to FIP.

In detail, cats were diagnosed with FIP if (1) FCoV RNA was detected by RT-qPCR (LABOKLIN GmbH & CO.KG, Bad Kissingen, Germany) or by RT-PCR (IDEXX Laboratories, Kornwestheim, Germany) in effusion in at least one body cavity in combination with (2) clinicopathological abnormalities and alteration in laboratory parameters considered typical for FIP [21,22,23]. Abdominal and/or pleural effusions were confirmed with abdominal and thoracic ultrasonography. A minimal body weight of 2 kg (AE Adam MTB 20 baby scale, Felde, Germany) was necessary to comply with feline guidelines for blood volume sampling according to the Gesellschaft für Versuchstierkunde—Society of Laboratory Animal Science [24]. FIV infection and progressive FeLV infection were ruled out before inclusion with a point-of-care test (SNAP Combo Plus FeLV-Ag/FIV-Antibody test, IDEXX Laboratories, Kornwestheim, Germany). 

Forty cats (31 male, 9 female) fulfilled the inclusion criteria and were recruited for the study (Table 2 and Table S1). The age of the 40 cats ranged from 5.1 to 116.3 months (median 13.5 months); 17 cats (42.5%) were younger than one year. The predominant breed (16/40; 40%) was Domestic Short Hair (DSH), followed by British Short Hair (BSH) (8/40; 20%); the other cats belonged to different breeds (16/40; 40%) (Table 2). The majority of cats were male (31/40; 77.5%), with 20 of them being neutered. Of the female cats (9/40; 22.5%), two were neutered. There was no significant difference regarding signalment and location of effusion between the two groups (Table 2). Of the 40 cats, 30 lived with one to up to four partner cats in the same household. The partner cat of cat 26 also developed FIP and subsequently was included in the study (cat 37).

### 2.4. Monitoring of Clinical and Laboratory Parameters

On day 1 prior to the administration of GS-441524, a comprehensive assessment involving a detailed medical history, thorough physical examination, abdominal ultrasonography, detailed cardiologic (including electrocardiogram, echocardiography, and in cases of suspected myocarditis, measurement of cardiac troponin I (cTnI)) and neurologic examination, as well as hematology and serum biochemistry were performed on all cats upon entry into the study. Urinalysis was performed if cystocentesis was possible (not performed on cats with thrombocytopenia, uncooperative cats, or small bladder size). Laboratory analyses assessed hematological and clinical chemistry parameters including symmetric dimethylarginine (SDMA), SAA, and AGP. Additionally, viral RNA loads in blood, effusion, and feces (voided fecal samples or fecal swabs), as well as serum anti-FCoV antibodies were measured. Subsequently, all clinical examinations and laboratory parameters were monitored throughout hospitalization and on each scheduled follow-up visit (days 14, 28, 42, 56, and 84) following a predetermined schedule (Table 1). In the case of severe anemia, it was not possible to collect enough blood for all analyses. Initially, daily abdominal and/or thoracic ultrasonography were performed and whenever enough effusion was present, abdominocentesis and/or thoracocentesis were performed. The volume of effusion was classified into four different grades: grade 0 (no effusion) to 3 (massive effusion). Samples were stored at –80 °C for the quantification of viral RNA loads.

The cats’ health and quality of life were assessed by the Karnofsky’s score modified for cats by Hartmann and Kuffer (1998). This scoring system employs a classification ranging from 0% (death) to 100% (normal health) [25]. The reference interval for the Karnofsky’s score in normal healthy cats was defined as 90–100%. 

Hematological analyses were conducted using automated analyzers (Cell-Dyn 3500, Abbott Laboratories, Chicago, IL, USA and ProCyte Dx, IDEXX Laboratories Inc., Westbrook, ME, USA). A manual differential blood count was performed on blood smears using Haema Quik staining/Diff-Quik staining. Serum biochemical parameters were assessed by an automated analyzer (Hitachi 911, Roche, Grenzach-Wyhlen, Germany). Symmetric dimethylarginine was quantified at IDEXX Diavet AG (Bäch, Switzerland) utilizing a high-throughput immunoassay. Serum amyloid A was examined through a latex agglutination turbidimetric immunoassay reaction (LZ Test SAA, Eiken Chemical Co., Ltd., Tokyo, Japan) on a cobas^®^ c 501 clinical chemistry analyzer (Roche Diagnostics AG, Rotkreuz, Switzerland). Reference interval: 0–3.9 mg/L [26]. Alpha-1-acid-glycoprotein was measured by a spatial proximity analyte reagent capture luminescence (SPARCL^TM^) assay on the VetBio-1 analyzer. Reference interval: <567 µg/mL [27]. Cardiac troponin I was measured by chemiluminescence using Siemens^®^ ADVIA Centaur XP (IDEXX Laboratories, Kornwestheim, Germany). Urinalysis was performed using automated analyzers (VetLab UA Analyzer and SediVue Dx Urine Sediment Analyzer, IDEXX Laboratories Inc., Westbrook, ME, USA). 

On day 7, cats were discharged from the hospital, unless their clinical condition required prolonged inpatient care. Subsequently, owners were responsible for the daily administration of the study drug, having received accurate instructions on how to apply the tablets. Owners were explicitly advised to contact the study team promptly in case of any issues. Throughout the remaining treatment period, owners were required to diligently monitor their cats at home, ensuring continuous observation, while also maintaining ongoing communication with the study team. Owners had to keep a daily diary to document activity, breathing rate, food/water intake, fecal/urine excretion, and body weight, ensuring the optimal dosing of the drug at all times. In addition, fecal consistency was monitored daily using the Purina fecal score, Nestlé Purina, St. Louis, MO, USA [28]. Outdoor access was not allowed to guarantee reliable drug administration and continuous surveillance.

### 2.5. Monitoring of FCoV Viral Loads and Anti-FCoV Antibodies

Feline coronavirus RNA loads were determined in blood samples on days 1, 3, 5, 7, 14, 28, 42, 56, and 84, in effusion samples as long as effusion was present and abdominocentesis or thoracocentesis, respectively, were possible, and in feces on days 1, 2, 3, 4, 5, 6, 7, and on days 14, 28, 42, 56, and 84. Samples were stored at –80 °C and sent in batches to the Clinical Laboratory of the Vetsuisse Faculty, University of Zurich, Switzerland, where they were analyzed by FCoV RT-qPCR as previously described [18,29]. Briefly, viral total nucleic acids (TNA) were extracted from 200 µL of effusion or 100 µL of ethylenediaminetetraacetic acid (EDTA) anticoagulated whole blood using the MagNa Pure 96 (Roche Diagnostics AG, Rotkreuz, Switzerland) and the DNA and Viral NA SV Kit (Roche Diagnostics, Indianapolis, IN, USA) according to the manufacturer’s instructions with an elution volume of 100 µL. Both voided fecal samples and fecal swabs were collected and processed as previously described [18]. A volume of 200 µL of processed fecal samples were used for TNA extraction as described above. With each batch of 96 extractions at least four negative controls (200 µL phosphate buffer saline (PBS; Gibco, Life Technologies Ltd., Paisley, UK)) were run in parallel to check for cross-contamination. An RT-qPCR assay was used to detect the FCoV 7b gene [30], modified according to Meli et al. [18], using AgPath-IDTM One-step RT-PCR kit (Applied Biosystems, Rotkreuz, Switzerland). The master mix consisted of 1X RT-PCR buffer, 1.0 µL Array Script reverse transcriptase and AmpliTaq Gold DNA polymerase, 300 nM forward primer (FCoV2), 900 nM reverse primer (FCoV1), 300 nM probe (FCoVp), and nuclease-free H_2_O was added to a final volume of 20 µL. All RT-qPCR assays were run with 5 µL of TNA for a final volume of 25 µL. The temperature profile included a step of reverse transcription for 10 min at 45 °C, followed by the initial denaturation and polymerase activation at 95 °C for 10 min and 45 cycles of 15 s at 95 °C, followed by 45 s at 60 °C. Fecal TNA samples were analyzed both undiluted and diluted at a 1:5 ratio in nuclease-free water to assess potential inhibition of RT-qPCR. Negative RT-PCR (molecular grade water) and extraction controls were included in parallel utilizing an ABI 7500 Fast instrument (Applied Biosystems). Additionally, a FCoV RNA standard curve was concurrently run to determine the viral RNA copy number as previously described [18].

Antibodies against FCoV were measured on days 1, 42, and 84. Serum samples were stored at –80 °C and subjected to analysis via an indirect immunofluorescence assay (IFA) following previously established protocols [31,32,33,34]. Samples were evaluated at dilutions of 1:25, 1:100, 1:400, and 1:1600. Each slide included a positive control (aliquoted serum sample from an anti-FCoV antibody-positive field cat) and a negative control (aliquoted serum from an anti-FCoV antibody-negative cat maintained under specific pathogen-free conditions). Results were evaluated in a fluorescence microscope (Leica LB 30T, Leica Microsystems, Wetzlar, Germany), with a 40× magnification, always by the same two laboratory technicians with more than 20 years of experience.

### 2.6. Data Analysis

Signalment of the two groups was compared using the Pearson Chi-squared test of independence. During the treatment, all variables were analyzed using mixed-effects linear models, with an individual cat as a random effect due to the repeated measures for an individual cat on multiple days. The following model assumptions were always checked: (1) the normality of residuals by the Shapiro–Wilk normality test, (2) the homogeneity of variances between groups by Levene’s test, and (3) the heteroskedasticity (constancy of error variance) by Breusch–Pagan test. In cases where assumptions were violated, a robust mixed-effects linear model (RLMER) was applied. RLMER computes weighted estimates via Design Adaptive Scale and thus solves heteroskedastic and non-normally distributed residuals by assigning lower weights to outliers and other contaminations. Moreover, model fit was assessed for both mixed-effects and robust mixed-effects models using coefficients of determination (R2), intraclass correlation coefficient (ICC), and RMSE. Models with a better fit were preferred. Bilirubin, SAA, AGP, fecal viral loads, and blood viral loads were log transformed due to a highly skewed distribution. All contrasts (differences) between particular days and duration categories were assessed after model fitting by the estimated least-squares marginal means (emmeans) with the Bonferroni *p*-value correction for multiple comparisons. Results with a *p*-value < 0.05 were considered statistically significant. All models were created and figures were produced using R Statistical language (version 4.3.1 (16 June 2023)). To visualize non-logarithmic and categorized FCoV viral RNA loads in blood, effusion, and fecal samples and serum anti-FCoV antibody titers of individual cats over time, heatmaps were created using Microsoft^®^ Excel version 16.83 (14031120).

## 3. Results

### 3.1. Cats at Inclusion

At the time of inclusion, 25/40 cats had abdominal, 5/40 pleural effusion, and 10/40 effusions in both body cavities (Table 2 and Table S1). In addition, two cats had neurologic signs during the first days of treatment (cats 13 and 21); cat 13 (short treatment group) had one generalized tonic–clonic seizure; cat 21 (long treatment group) showed hind limb weakness, positional vertical nystagmus, and developed multiple tonic–clonic seizures. Uveitis was diagnosed in one cat (cat 24, long treatment group). Myocarditis was diagnosed in four cats (cats 24, 38, 39, and 40; all four cats belonged to the long treatment group) by electrocardiogram, echocardiography, and cTnI measurement.

### 3.2. Efficacy of the Oral GS-441524 

Clinical remission was achieved between days 14 and 84 with a median of 28 days. All cats were monitored until day 168 after the treatment initiation and remained in complete remission. Within the first 42 days of treatment, 37/40 cats (92.5%; 19 in short, 18 in long treatment group) went into complete clinical remission. One cat (long treatment group) still showed signs of myocarditis on day 42 and was in partial remission. Two cats were euthanized during treatment (day 3 and day 31) due to secondary complications.

**Figure 3:**
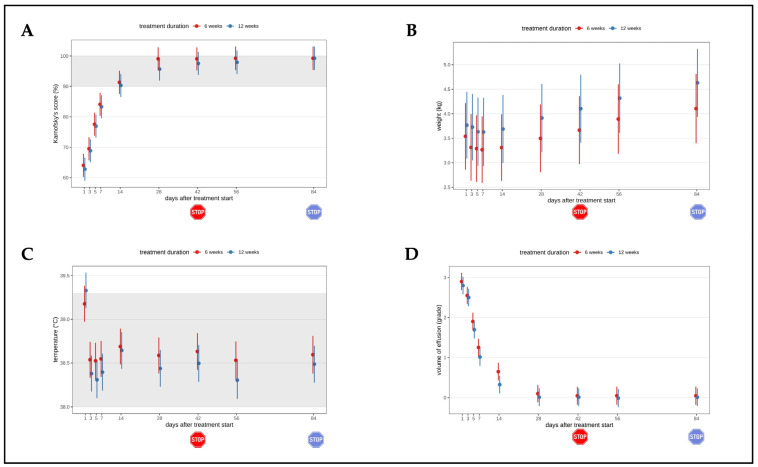
Timeline visualizing improvement of clinical parameters throughout the study course in both groups until day 84 of the study (comparison of treatment duration six versus twelve weeks). The red stop sign marks the end of treatment of the short treatment group; the blue stop sign marks the end of treatment of the long treatment group. Figures show average predictive values and 95% confidence intervals of each parameter. Grey shading marks the reference interval of the parameters. (**A**) Karnofsky’s score modified for cats. (**B**) Body weight. (**C**) Body temperature. (**D**) Volume of effusion subjectively evaluated during abdominal/thoracic ultrasonography and paracentesis (grades 0 (no effusion) to 3 (massive effusion)).

**Figure 4:**
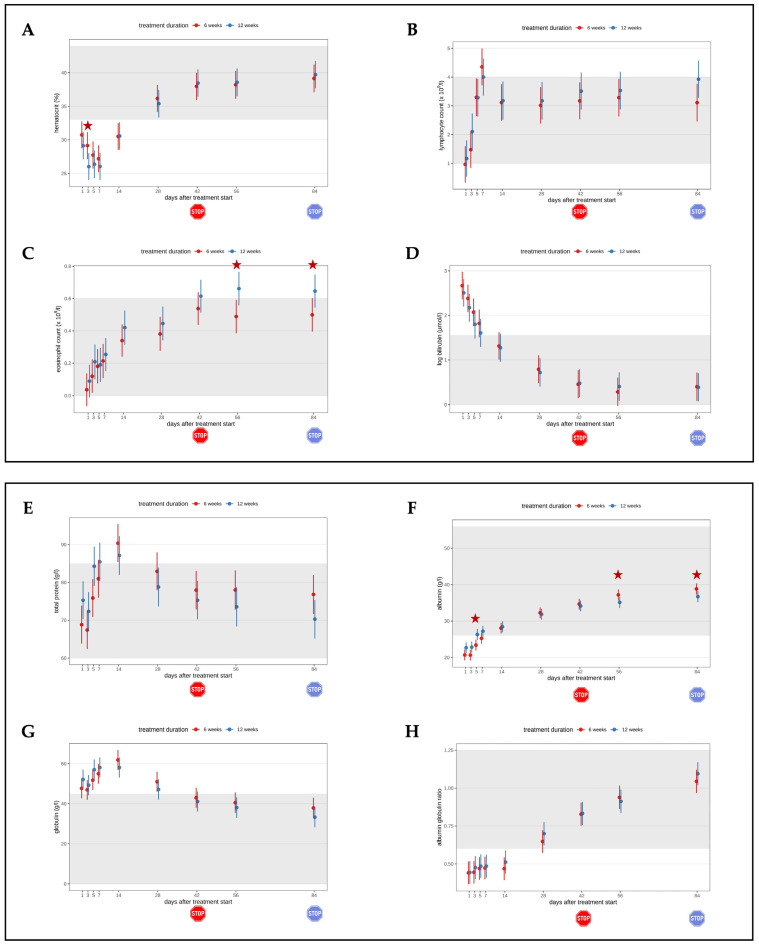

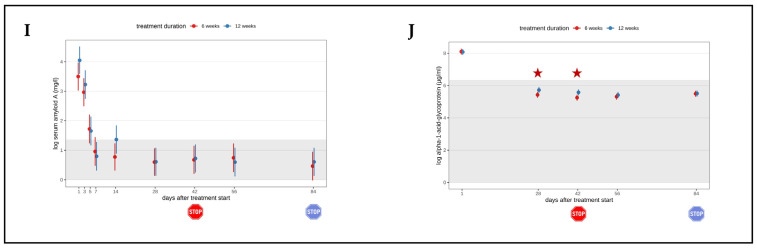
Timeline visualizing improvement of laboratory parameters throughout the study course in both groups until day 84 of the study (comparison of treatment duration six versus twelve weeks). The red stop sign marks the end of treatment of the short treatment group; the blue stop sign marks the end of treatment of the long treatment group. Figures show average predictive values and 95% confidence intervals of each parameter. Grey shading marks the reference interval of the parameters. Red asterisks mark significant differences (*p* < 0.05) of the parameters in the group comparison. (**A**) Hematocrit. (**B**) Lymphocyte count. (**C**) Eosinophil count. (**D**) Bilirubin concentration. (**E**) Total protein concentration. (**F**) Albumin concentration. (**G**) Globulin concentration. (**H**) Albumin/globulin ratio. (**I**) Serum amyloid A (SAA) concentration. (**J**) Alpha-1-acid-glycoprotein (AGP) concentration.

In all cats (with the exception of the two euthanized cats), the clinical, hematological, and clinical chemistry parameters typically altered in FIP cats [21,22,23] improved consistently and significantly during treatment in both groups (Figure 3 and Figure 4), with significant differences in the parameters between day 2 and day 42 when compared to day 1 (start of treatment). The Karnofsky’s score (Figure 3A) of the cats improved quickly within the first days of treatment, with significant changes compared to day 1 in all but the two euthanized cats, and was within the reference interval by day 28 at the latest. Most cats gained body weight rapidly (Figure 3B). Also, the body temperature normalized soon after treatment initiation in both groups (Figure 3C). In all cats, the volume of effusion decreased steadily (Figure 3D). Only three cats (cats 2, 4, 14) had accessible abdominal or pleural effusion on day 14. 

**Figure 5:**
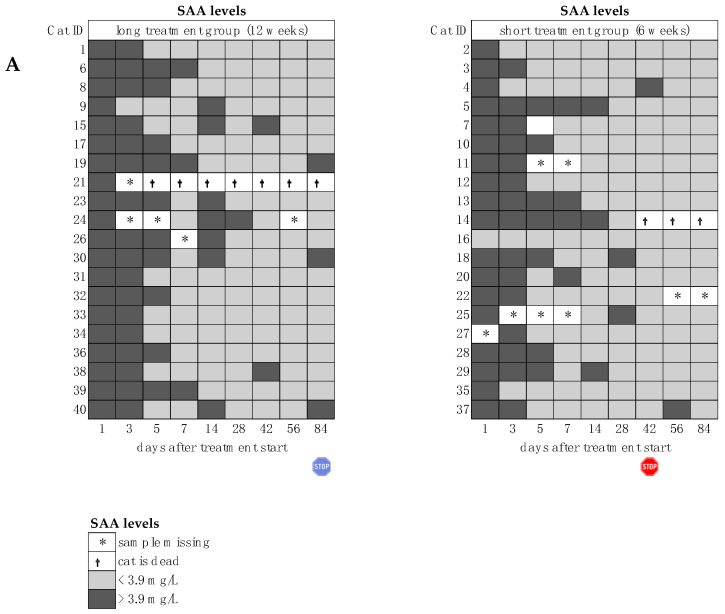

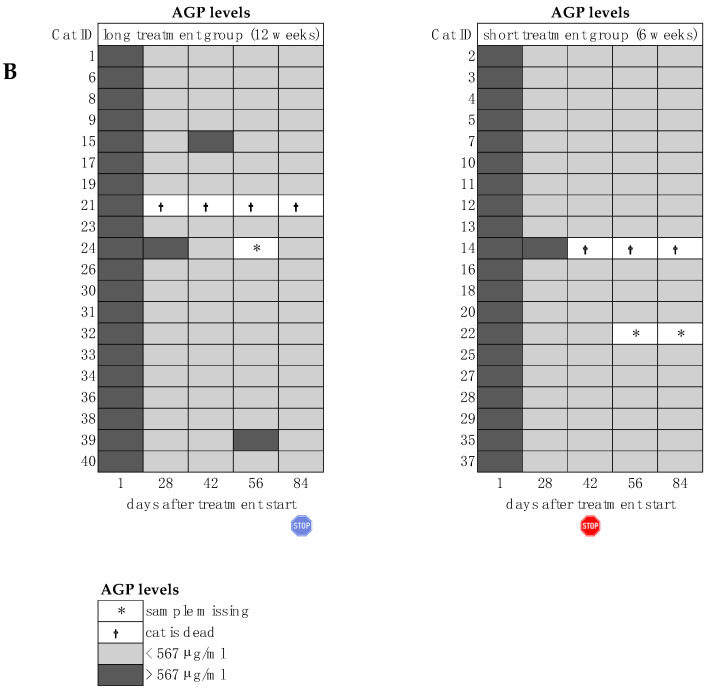
Heatmaps visualizing improvement of serum amyloid A (SAA) and alpha-1-acid-glycoprotein (AGP) concentrations throughout the study course in both groups until day 84 of the study (comparison of treatment duration six versus twelve weeks). The red stop sign marks the end of treatment of the short treatment group; the blue stop sign marks the end of treatment of the long treatment group. (**A**) SAA concentration. (**B**) AGP concentration. * Samples missing, e.g., due to severe anemia.

Hematological parameters also improved rapidly. Four cats received whole blood transfusions due to severe anemia during the first days of treatment. The hematocrit subsequently normalized in three of the four cats. One cat developed immune-mediated hemolytic anemia (diagnosed by autoagglutination and exclusion of blood parasites), was treated with prednisolone, and the hematocrit returned into the reference range by day 42. Also, lymphocyte and eosinophil counts, bilirubin, total protein, albumin, and globulin concentrations, and the albumin/globulin ratio improved rapidly (Figure 4). On day 1, 38 of 39 cats, in which SAA was measured, had increased concentrations (Figure 4I and Figure 5A). On day 28, at the latest, the SAA concentrations of all cats were within the reference interval. However, mild increases in SAA concentrations were observed intermittently. On day 1, all 40 cats had increased AGP concentrations (Figure 4J and Figure 5B). Alpha-1-acid-glycoprotein concentrations were within the reference interval in 37/39 cats by day 28. 

**Figure 6:**
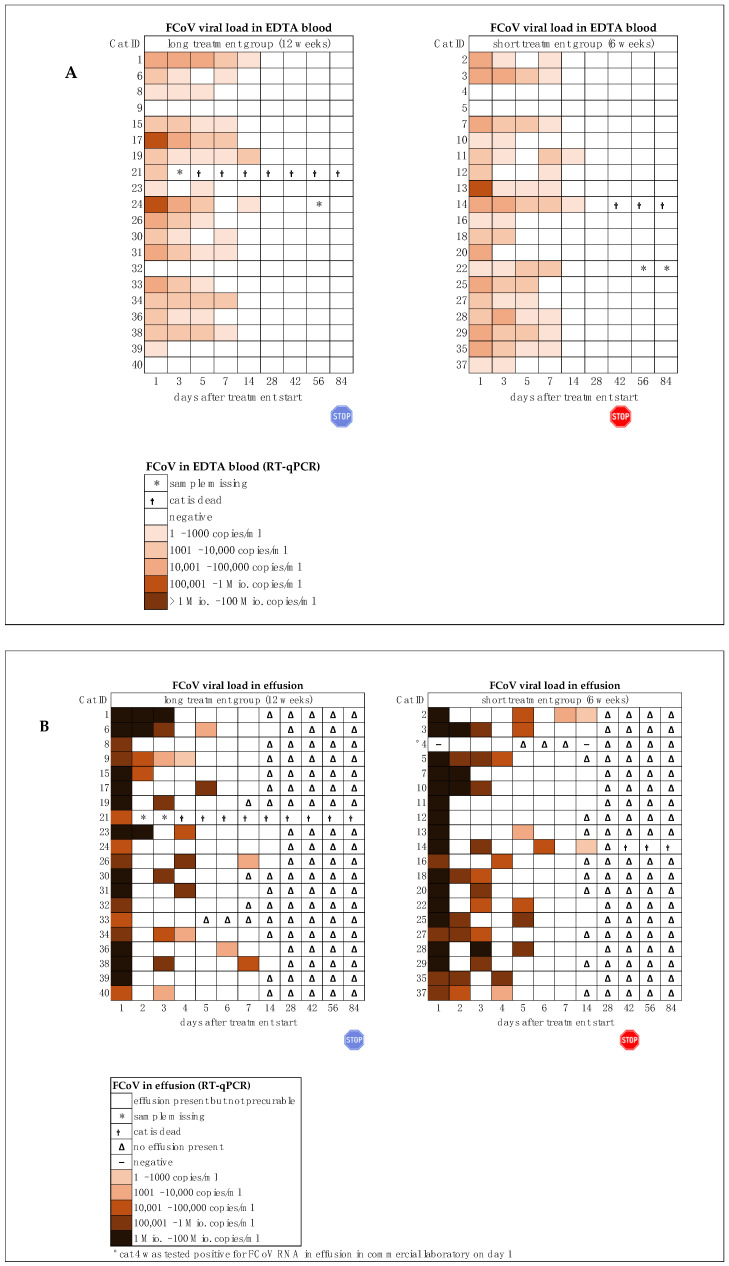

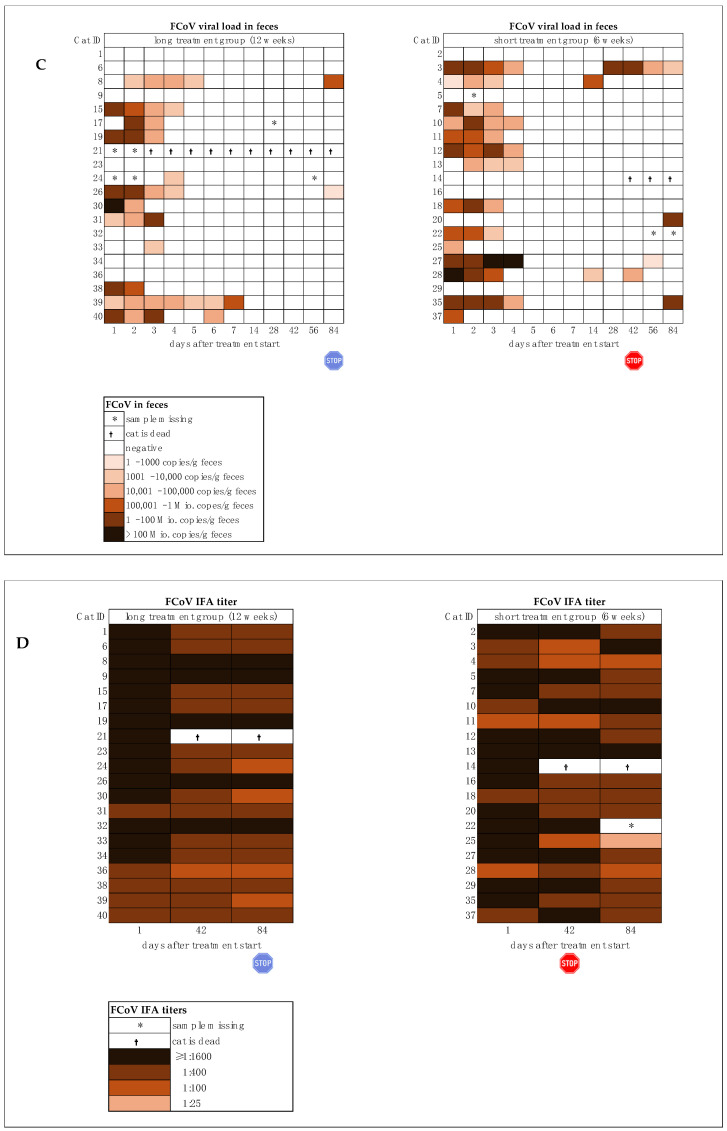
Feline coronavirus (FCoV) viral RNA loads in blood, effusion, and fecal samples and serum anti-FCoV antibody titers throughout the study course in both groups until day 84 of the study (comparison of treatment duration six versus twelve weeks). The red stop sign marks the end of treatment of the short treatment group; the blue stop sign marks the end of treatment of the long treatment group. (**A**) FCoV RNA loads in EDTA anticoagulated blood. (**B**) FCoV RNA loads in effusions. (**C**) FCoV RNA loads in feces. (**D**) Serum anti-FCoV antibody titers. FCoV RNA loads were determined by quantitative reverse transcriptase polymerase chain reaction (RT-qPCR). Antibody titers were determined by indirect immunofluorescence assay (IFA). * Samples missing, e.g., due to severe anemia.

Viral RNA was detected in the blood of 35/40 cats before treatment; cats 4, 5, 9, 32, and 40 tested negative (Figure 6A). The viral RNA loads in blood on day 1 ranged from 94 copies/mL (lowest measured viral RNA load in blood, cat 37) to 427,973 copies/mL EDTA blood (highest measured viral RNA load in blood, cat 13). Blood viral RNA loads decreased remarkably with a significant difference by day 5 compared to day 1 in both groups. Viral RNA was no longer detected in the blood of any cat by day 28, indicating that all cats had cleared viremia. Viral RNA was detected in effusions of all 40 cats before treatment (inclusion criteria). However, in one cat (cat 4), viral RNA in effusion was only found in a commercial laboratory (Figure 6B), and the cat was negative by RT-qPCR in the laboratory of the Vetsuisse Faculty, University of Zurich, in effusion at the start of treatment. Viral RNA loads in effusion on day 1 ranged from 28,073 copies/mL (lowest measured viral RNA load in effusion, cat 24) to >45 million copies/mL (highest measured viral RNA load in effusion, cat 3). Viral RNA loads in effusion decreased steadily with a significant difference by day 3 compared to day 1 in both groups. In all cats, the volume of effusion decreased rapidly (Figure 3D). On day 14, only three cats (cats 2, 4, 14) had accessible abdominal or pleural effusion. In cat 4 (short treatment group), no viral RNA was detectable in abdominal effusion, while in cats 2 and 14 (both short treatment group), viral RNA was still detectable. On day 18, one cat (cat 14, short treatment group) had accessible pleural effusion; no viral RNA was detectable in the effusion sample. In 21/38 of the treated cats (55.3%), (in two cats, fecal samples were not available on day 1), fecal FCoV RNA shedding was detectable before treatment initiation (Figure 6C). In all 21 cats that shed FCoV on day 1, fecal virus shedding ceased during treatment. Furthermore, five cats (cats 8, 13, 17, 24, and 33) that initially showed no fecal virus shedding in voided fecal samples or swabs became RT-qPCR-positive between days 2 and 4. In 10 of 26 cats, viral RNA loads in feces initially increased during treatment. In all 26/40 cats that were shedding FCoV in the first days of treatment (days 1–4), fecal virus shedding had ceased after day 7 at the latest. From day 14, 8/26 cats restarted fecal virus shedding intermittently. On day 1, viral loads in voided fecal samples ranged from 186 copies/g feces (cat 4) to almost 628 million copies/g feces (cat 28). In fecal swabs, viral loads ranged from as low as 510 copies per swab (cat 27 on day 56) to as high as 1.1 million copies per swab (cat 3 on day 1). At the onset of the study, all cats had detectable anti-FCoV antibody titers (Figure 6D). In 31/40 cats, a high titer of ≥1:1600 was detected. Those high anti-FCoV antibody titers (≥1:1600), but no viral RNA in feces were observed in 16/38 cats on day 1; nine of these cats belonged to the long treatment group and seven to the short treatment group. In 25 of the 38 surviving cats, the antibody titers declined until day 84. A high anti-FCoV antibody titer (≥1:1600) was maintained in 6/38 cats throughout the 84 days. An increase in anti-FCoV antibody titers was observed in 5/38 cats. Two of these five cats (cats 3 and 28) had ceased fecal virus shedding during treatment but later tested FCoV RNA-positive in feces again. 

### 3.3. Comparison between Short and Long Treatment 

During the first phase (day 1 to day 42) all cats were receiving treatment. On day 1 (inclusion), no significant differences regarding clinical and laboratory parameters were observed between the cats of the short and the long treatment group (Figure 3 and Figure 4). On day 3, cats in the long treatment group had significantly lower hematocrit levels compared to the cats in the short treatment group (*p* = 0.03). On day 5, cats in the short treatment group had significantly lower albumin concentrations compared to the cats in the long treatment group (*p* = 0.01). On days 28 and 42, cats in the long treatment group showed significantly higher AGP concentrations (*p* = 0.02 *p* = 0.01). Viral RNA loads in effusions decreased steadily in both groups with a significant difference in the group comparison on day 4; cats in the short treatment group had significantly higher viral RNA loads in effusion (*p* = 0.03) (Figure 6B). No other differences in viral parameters (viral loads in blood and feces and anti-FCoV antibody titer) were observed between the groups in the first phase.

During the second phase, cats of the short treatment group had terminated their treatment; only cats in the long treatment group were still receiving GS-441524. On days 56 and day 84, cats in the long treatment group showed a significantly higher eosinophil count compared to the cats in the short treatment group (*p* = 0.02, *p* = 0.04). On days 56 and 84, 4/18 cats (22.2%) in the short treatment group had eosinophilia. On day 56, 9/18 cats (50%) and on day 84, 11/19 cats (57.9%) in the long treatment group had eosinophilia. On days 56 (*p* = 0.048) and 84 (*p* = 0.04), cats in the long treatment group showed significantly lower albumin concentrations compared to the cats in the short treatment group; however, albumin concentrations of all cats were within the reference interval. 

During the second phase, no significant differences regarding viral loads in blood, effusion, and feces nor regarding anti-FCoV antibodies were observed. In all 26/40 cats that were shedding FCoV in the first days of treatment (days 1–4), fecal virus shedding ceased after day 7 at the latest. From day 14, 8/26 cats restarted fecal virus shedding intermittently. Of these eight cats, two belonged to the long treatment group and shed FCoV on day 84 and six belonged to the short treatment group; three cats of the latter restarted fecal virus shedding after day 42 (after the end of treatment). Anti-FCoV antibody titers decreased in 25/38 surviving cats until day 84; 14 cats were in the short treatment group and 11 cats were in the long treatment group. There was no significant difference in the group comparison. In 5/38 cats, an increase in anti-FCoV antibody titers was observed during treatment; all five of these cats were in the short treatment group.

### 3.4. Adverse Events during Oral GS-441524 Treatment in Cats with FIP 

During treatment with GS-441524, a few adverse events were observed (Table S1). Diarrhea was seen in 25/40 cats; in 5/25, diarrhea was severe, equivalent to a Purina fecal score of 7 [28], and was treated with probiotics and fluid therapy. In 2/5 cats, >5% red blood cells containing Heinz bodies were observed (6.3% and 19%, respectively). Lymphocytosis was seen in 27/40 (67.5%) cats. Some cats (26/40; 65%) developed mild eosinophilia between days 1 and 84 (<2.0 × 10^9^/L, reference interval: 0–0.6 × 10^9^/L). An increase in liver enzyme activity was noted in 24/40 (60%) cats between days 1 and 84, but this was mostly mild to moderate; four cats were treated with silymarin, three cats were also treated with S-adenosyl-methionine (SAMe), and liver enzyme activity normalized in most cats until day 84. On day 168 (final recheck), 3/37 cats still showed increased liver enzyme activity. A mild increase in SDMA above the reference interval was seen in 25/40 (62.5%) cats. 

### 3.5. Postmortem Findings in Cats 21 and 14 

Two cats were euthanized on day 3 and day 31 due to secondary complications of FIP. Both cats had a complete postmortem examination within 24h following euthanasia. During necropsy, samples were collected from virtually all organs and tissue types as well as from intestinal contents of the duodenum, jejunum, ileum, caecum, and colon. Tissue sections from all areas (including tonsils, multiple lymph nodes, spleen, mesenterium, stomach, duodenum, jejunum, ileum, cecum, colon, rectum, kidneys, liver, pancreas, lungs, heart, brain, and spinal cord) were investigated by immunohistochemistry for FCoV antigen expression using FIPV3-70 monoclonal antibody (MCA2194) (Bio-Rad Laboratories GmbH, Feldkirchen, Germany), as described previously [16]. Two brain sections from a cat with confirmed FIP affecting the central nervous system were used as the positive control. Negative controls included substitution of the primary antibody serum by phosphate-buffered saline (PBS) and the use of an irrelevant mouse monoclonal antibody (Bo-18) as the primary antibody. 

Cat 21 (long treatment group) had signs of neurologic disease on admission (pelvic limb weakness and postural reaction deficits, positional vertical nystagmus), severe hypoglycemia, experienced multiple seizures on days 2 and 3 of treatment, and subsequently never regained full consciousness. The cat also showed signs of sepsis. GS-441524 was administered by an oral–gastric tube. On day 3, the cat had a cardiopulmonary arrest and despite resuscitation, the owner opted for euthanasia on day 3 of treatment. 

**Figure 7:**
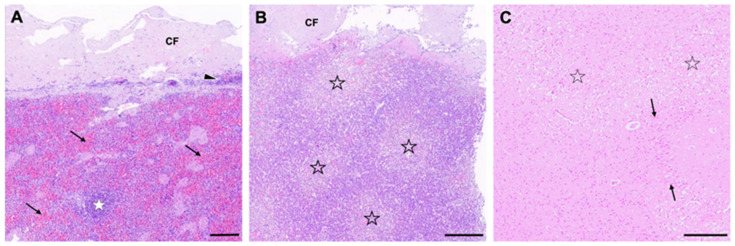
Histopathological characteristics of cat 21. (**A**) Spleen with capsular fibrosis (CF) on top of lymphoplasmacytic infiltrates (arrowhead) consistent with chronic perisplenitis. Occasional lymph follicles (white asterisk) and congestion are seen (arrows). (**B**) The liver likewise presented with an extensive capsular fibrosis (CF). There was a significant centrolobular degeneration suggestive of hypoxic changes (asterisk). (**C**) The brain showed multifocal spongiosis within cerebral cortex (asterisk) if compared to well-delineated unaffected segments (arrows). This change was compatible with malperfusive changes. Scale bars: (**A**) 235 µm; (**B**,**C**) 350 µm. (**A**–**C**): HE-FFPE.

In the necropsy, severe chronic lymphoplasmahistiocytic as well as fibroplastic peritonitis involving serosal surfaces of virtually all visceral organs, liver, and spleen (Figure 7) were present and extended into the tunica muscularis of the intestine, in terms of an interstitial leiomyositis. Compatible with the clinically reported cardiopulmonary arrest, there was an alveolar pulmonary edema as well as a centrilobular hepatocellular vacuolar degeneration (Figure 7B). In the same vein, the brain of cat 21 revealed multifocal well-delineated spongy changes with neuronal degeneration throughout the neocortex, most consistent with subacute ischemic changes (Figure 7C). 

None of the immunohistochemical specimens showed immunopositivity for FCoV antigen in IHC. 

**Figure 8:**
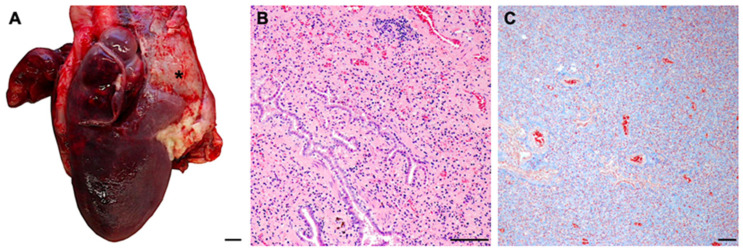
Pathological findings in cat 14. (**A**) Lateral aspect of the right lung and pericardium (*). The lung presented with diffuse, marked dark-red discoloration and diffuse congestion. The apex lobe was focally attached to the pericardium. Scale bar = 1 cm. (**B**) Lung histology. There was a diffuse loss of lung architecture with diffuse and marked interstitial fibrosis, compression of bronchioles, and a mixed-cellular, predominantly mononuclear round cell infiltration. Lung, HE-FFPE. Scale bar = 100 µm. (**C**) Lung histology. Masson’s trichrome demonstrates the diffuse and marked interstitial fibrosis. Lung, MT-FFPE. Scale bar = 100 µm.

Cat 14 (short treatment group) was euthanized on day 31 of treatment due to severe and non-controllable dyspnea. This cat had shown a remarkable improvement in laboratory parameters and a reduction in viral loads in blood and effusion. In necropsy, cat 14 presented with a marked interstitial lung fibrosis and multifocal coalescing chronically active inflammatory lesions, compatible with a severe restrictive pneumopathy, which explains the final clinical presentation (Figure 8). FIP-associated changes were restricted to a mixed leukocytic fibrinous to fibroplastic pleuritis. None of the immunohistochemical specimens, however, showed immunopositivity for FCoV antigen in IHC.

## 4. Discussion

This prospective randomized controlled study clearly demonstrated that a treatment duration of 84 days (12 weeks)—as currently recommended by publications, guidelines, and by social media groups—is not necessary for cats with FIP. A shorter treatment duration of 42 days (six weeks) with oral GS-441524 also leads to sustained complete remission in cats with FIP. One of the inclusion criteria was the presence of effusion in at least one body cavity. Nevertheless, two of the surviving cats (one in the short and one in the long treatment group) additionally had neurologic or ocular signs that resolved during treatment with GS-441524. Both cats remained in complete remission during the observation period (up to 168 days after treatment initiation). Therefore, treatment with oral GS-441524 for 42 days (six weeks) is likely sufficient to achieve complete remission not only in FIP cats with effusion but also with neurological and/or ophthalmological symptoms. Further studies are necessary to support this assumption.

The previously recommended treatment period of 84 days that has been used in many studies was based on positive outcomes observed in these earlier studies [3,5,8]. In the present study, the majority of the cats treated for 42 days and for 84 days (19/20 cats in both groups) exhibited a rapid improvement in general well-being and laboratory parameters, as well as a reduction in viral loads. 

Cats were followed closely until day 168 (24 weeks), and all 38 cats remained in complete remission. There were no significant differences in clinical relevance observed between the two groups. No signs of relapse or any clinical or laboratory changes typically altered in FIP cats occurred in the short treatment group. Only one cat (cat 24, long treatment group) still showed signs of myocarditis on day 42 and was still receiving cardiovascular therapy at that time point. On day 84, the myocardial function had returned to normal with no further medication necessary. This cat was in the long treatment group and received GS-441524 beyond day 42, and it remains unclear whether prolonged administration of GS-441524 was necessary in this specific cat to achieve complete remission. It is, however, more likely that residual cardiac lesions of the previous disease FIP required regeneration without the presence of any FCoV. The cat was (like all others) negative in blood RT-PCR at that time point. This assumption is also supported by the fact that cat 14 that was euthanized on day 31 still had severe morphologic changes in its lung but no FCoV was detected by IHC. Thus, the virus was long eliminated but the organ changes still remained. The survival rate in this study was 95%, and this is similar or better to what has been described in other studies, such as 77.4% [3], 75% [5], 100% [8], and 55.6% [13], in which cats were treated for at least 84 days. Thus, the survival rate of the cats in the short treatment arm of the present study was identical to what has been described in other studies, and the two cats that were euthanized had their euthanasia early at a time where they were still treated anyway.

Minor differences between the two groups were observed regarding selected laboratory parameters, including hematocrit (Figure 4A), eosinophil count (Figure 4C), albumin (Figure 4F), and AGP concentrations (Figure 4J). During the second phase, in which cats of the short treatment group had already terminated their treatment, cats that were still being treated with GS-441524 had a significantly higher eosinophil count (Figure 4C) than cats in which the treatment had been stopped. In previous studies, the development of eosinophilia during FIP treatment has already been described [8,10] and occurred in 61.1% [8] and 52% [10] of cats during treatment. In human medicine, eosinophilia has been proposed as a marker for favorable outcomes [35,36,37]. Interestingly, in the present study, it seems like eosinophilia was directly induced by the drug, as it was only present (in a majority of cats) as long as the cats received the drug. Thus, it is less likely a sign of regeneration. Possibly, GS-441524 activated the complement system, thereby chemotactically attracting eosinophils. Additional investigation of this interesting finding in cats treated with GS-441524 is needed; it would be crucial to know if healthy cats (without FIP) develop eosinophilia as well when being treated with GS-441524. A significant difference between groups also occurred in albumin concentrations on days 56 and 84. Cats in the short treatment group had significantly higher albumin concentrations than cats in the long treatment group, but this was considered irrelevant since all albumin concentrations were within the reference interval. Some minor significant differences (in hematocrit, albumin, and AGP) between the two groups were already observed during the first phase when both groups were still receiving treatment. Therefore, these differences were not considered relevant.

Viral RNA loads in blood decreased significantly during the first five days of treatment in both groups. None of the cats was FCoV RNA-positive in blood from day 28 on, indicating that all cats had cleared detectable viremia. In the current study, clearance of viremia was used as an important parameter to demonstrate treatment efficacy. FCoV viremia, when it occurs, is transient, peaking around 7 to 14 days post-infection and is subsequently declining [38,39]. Consequently, by the time clinical signs of FIP emerge, viremia cannot always be detected. In previously published studies, RT-PCR on blood samples often yielded negative results [22]. However, in the present as well as in previous studies of the authors’ research group, using well-standardized blood sampling, storing, and shipping techniques and a highly sensitive RT-PCR, such as the one used in the present study which was performed in the Clinical Laboratory of the Vetsuisse Faculty, University of Zurich, Switzerland (Prof. Hofmann-Lehmann), FCoV RNA could be detected in the blood of 83.3% [8] to 87.5% of cats with FIP. This indicates that FCoV viremia can persist longer in cats (before being treated) than previously believed.

Viral RNA loads in effusions also decreased steadily in both groups with a significant difference between the groups only once on day 4 when cats in the short treatment group had significantly higher viral loads in effusions (Figure 6B). This difference could indicate that cats in the short treatment group were more severely affected. However, cats with higher viral loads also showed a rapid improvement in clinical and laboratory parameters, indicating that high virus loads are not necessarily an indicator of more severe disease. In one cat (cat 4), viral RNA in effusion was only found in a commercial laboratory by RT-qPCR, and the cat was negative by RT-qPCR in the Clinical Laboratory of the Vetsuisse Faculty, University of Zurich, in effusion at the start of treatment. However, the volume of effusion obtained by abdominocentesis was only 50 µL; potentially, this volume was insufficient to detect the virus. The cat fulfilled all inclusion criteria and showed remarkable improvement in clinical and laboratory parameters; therefore, the cat was not excluded.

It should be noted that in the present study, viral RNA loads in blood became undetectable by day 28, which is later than in the initial study performed by the same research group [8], in which viral RNA loads in blood had already become undetectable by day 14. In that former study, all cats were in complete remission, even one year after the initiation of treatment [17]. The GS-441524 used in the previous study was produced by Mutian Life Sciences Limited and in addition to GS-441524 contained herbal preparations. Perhaps, in the former study, the additional components in the GS-441524 tablets played a role in enhancing the GS-441524 effect or acted as an immunostimulant. In the former study, GS-441524 was given at a dosage of 5 mg/kg or 10 mg/kg q24h PO (in case of neurologic/ophthalmologic signs) according to the manufacturer. However, the analysis of the multicomponent drug by liquid chromatography and mass spectrometry revealed that the tablets contained more than double the amount of GS-441524 than stated by the manufacturer (personal communication J. Horak); thus, cats likely received more than indicated by the manufacturer. In the present study, a dosage of 15 mg/kg q24h PO was chosen based on that prior study [8] for all cats, independently of whether they had neurologic and/or ophthalmologic signs or not. This was based on the fact that in most cats, FCoV also invades the brain even without visible neurologic signs [40]. It is well known that FCoV commonly disseminates from body cavities to the brain, and failure to achieve long-term disease remission can be associated with the occurrence of neurologic disease in cats that initially did not show any signs of brain or spinal cord involvement [40]. Therefore, for cats both with and without neurological signs, a uniform dosage of 15 mg/kg q24h PO was chosen.

Fecal virus shedding ceased during treatment in all 21 cats that initially exhibited shedding; however, 8/40 cats later tested positive again, presumably due to reinfection by a partner cat. Of these eight cats, five restarted shedding viral RNA during treatment with GS-441524. The remaining three cats had already terminated treatment on day 42. Based on these results, it can be concluded that cats can shed FCoV even while undergoing treatment with GS-441524. This clearly shows that cats without FIP should not be treated with GS-441524 only to terminate FCoV shedding, as this will not be effective in the long term and might lead to resistance development.

In 25/38 surviving cats, the anti-FCoV antibody titers declined until day 84, but in 5/38 cats, an increase in anti-FCoV antibody titers was observed during the observation period. All five of these cats were in the short treatment group, but 3/5 were still receiving treatment when the increase in anti-FCoV antibody titers occurred. At the time of the increase, 2/5 cats (cats 3 and 28) were shedding FCoV in feces. Thus, it is likely coincidental that an increase in anti-FCoV antibody titers was only observed in the short treatment group.

Cat 14 was euthanized on day 31 due to severe pulmonary disease resulting in persistent severe dyspnea. This cat had shown a remarkable improvement in laboratory parameters and a reduction in viral RNA loads. The cat was FCoV RNA-negative in blood by day 28, in effusion by day 18, and in postmortem examination, no FCoV was detected by IHC in any of the investigated tissues. Apparently in this cat, GS-441524 was effective in eliminating the virus. It is known that FIP can induce pyogranulomatous pneumonia, yet the impact of FIP on the respiratory system remains inadequately understood. The lungs are not typically regarded as a primary target of FIP; their involvement is often viewed as a result of systemic disease, generally presenting with localized lesions in the parenchyma beneath the pleura [41]. The origin of the pulmonary changes in this cat is unclear, but it is possible that they occurred secondarily due to bacterial infection caused by thoracentesis.

The other euthanized cat (cat 21) was in a severe clinical condition with neurologic signs upon admission and suffered from recurrent seizures, hypoglycemia, third-degree heart block, and experienced a cardiopulmonary arrest. It is likely that oral treatment might not be the ideal application route for cats in such moribund conditions with reduced gastrointestinal motility. In such cases, intravenous GS-441524 or remdesivir formulations, potentially followed by oral GS-441524, might be advantageous and achieve higher plasma levels. Unfortunately, these treatment options were not available.

The adverse events of GS-441524 observed in the present study during treatment were not severe, and the drug was well tolerated in all cats. Despite the overall promising results, GS-441524 is still not licensed in many countries. GS-441524 was patented in 2009 by Gilead Sciences, Inc., Foster City, CA, USA, which has refused to release the patent ever since. As an exception, in some countries, such as the United Kingdom, Australia, and more recently in Canada and the United States, cats can be legally treated with GS-441524 using unlicensed products. GS-441524 is legally manufactured and distributed by BOVA Specials, London, UK, in a strictly controlled manner. However, since it is unlicensed, it cannot be imported by veterinarians to Germany or most other European countries unless in the context of a study officially approved by the respective governing body. Therefore, many cat owners rely on social media platforms to obtain GS-441524 preparations from non-controlled underground manufacturers who are usually based in China [6]. Thus, in many published case reports, compounds containing uncertain dosages of GS-441524 provided by non-controlled productions and with undefined medical and legal risks were used [42]. These social media platforms currently strictly advise an 84-day treatment. Thus, it is very important that the present study, indicating that such long treatment is not necessary, has an impact on cats treated worldwide. Shorter treatment means fewer adverse effects in treated cats, less stress for cats, and a lower financial burden for owners, thus hopefully contributing to more cats being treated worldwide. Considering the results of the current study, it could of course be discussed whether an even shorter treatment duration would be sufficient. In the first study on GS-441524 specific pathogen-free cats experimentally infected with FIP-causing FCoV and clinical signs of FIP were treated with GS-441524 for only 14 days, and all cats were in remission at the time of publication [2]. However, an experimental situation cannot necessarily be applied to the field. Therefore, six weeks (42 days) was considered a safe first approach. Knowing the results of the present study now, it might be considered to even further reduce the treatment duration in future studies.

Limitations of this study included the inability to collect all samples on every day of the study. This was primarily attributed to the initial severe anemia observed in several cats in the beginning of the study, resulting in an insufficient volume of blood being safely obtainable. Furthermore, some cats needed blood transfusions, which might have altered some parameters. 

## 5. Conclusions

In conclusion, this prospective randomized controlled study demonstrated that treatment with oral GS-441524 for 42 days (six weeks) is sufficient to achieve complete remission in cats suffering from FIP and to maintain this remission up to 168 days (24 weeks). A longer treatment duration of 84 days, as currently recommended, is therefore obviously not necessary, at least in cats stable by day 42. There were no significant differences in clinical relevance observed between the cats receiving GS-441524 for 42 days compared to cats treated for 84 days regarding improvement in clinical and laboratory parameters as well as viral loads. A shorter treatment duration reduces risk of potential adverse effects, costs of treatment, and the potential risk for the development of resistant viruses. 

## Figures and Tables

**Table 1:** Monitoring schedule of the 40 cats during hospitalization (days 1 to 7) and on-site follow-up examinations (days 14, 28, 42, 56, 84).

Day	Physical Examination ^#^	Abdominal and Thoracic Ultrasonography ^#^	Detailed Cardiologic and Neurologic Examinations ^#^	Hematology ^#^, Clinical Chemistry ^#^, Including SDMA ^1^, SAA ^2^, AGP ^3^, and Urinalysis *	Viral RNA Loads (RT-qPCR) in Effusion, Blood, and Feces	Anti-FCoV Antibodies
**1**	x	x	x	x	x	x
**3**	x			x	x	
**5**	x	x		x	x	
**7**	x	x		x	x	
**14**	x	x		x	x	
**28**	x	x		x	x	
**42**	x	x	x	x	x	x
**56**	x	x		x	x	
**84**	x	x	x	x	x	x

^1^ Symmetric dimethylarginine, ^2^ Serum amyloid A, ^3^ Alpha-1-acid-glycoprotein (samples taken on each scheduled day, measurements conducted on days 1, 28, 42, 56, and 84), * performed when cystocentesis was possible, ^#^ in case of any abnormalities, these examinations or parameters were assessed more often.

**Table 2:** Description of the study cohort (*n* = 40), including signalment and location of effusion.

		Total Number of Cats	Cats with 42 Days of Treatment	Cats with 84 Days of Treatment	*p*-Value *
**Number of Cats**		40	20	20	
**Breed**	DSH ^1^	16	11	5	0.13
	BSH ^2^	8	3	5	0.48
	other breeds ^3^	16	6	10	0.32
**Age**	<1 year	17	10	7	0.81
	>1 year	23	11	12	0.83
**Sex**	female intact	7	5	2	0.26
	female neutered	2	1	1	1.00
	male intact	11	3	8	0.13
	male neutered	20	11	9	0.65
**Location of Effusion**	abdominal	25	14	11	0.55
	pleural	5	3	2	0.65
	abdominal/pleural	10	3	7	0.21

^1^ Domestic Short Hair; ^2^ British Short Hair; ^3^ Maine Coon (mix) (4/16); Siamese (mix) (2/16); Domestic Longhair mix (1/16); DSH/BSH mix (1/16); Exotic Short Hair (1/16); Holy Birman (1/16); Oriental Short Hair (1/16); Ragdoll (1/16); Russian Blue Mix (1/16); Scottish Fold (1/16); Scottish Straight (1/16); Somali (1/16); * *p*-values for a potential difference between groups were determined using the Pearson Chi-squared test.

## Data Availability

The authors confirm that the datasets analyzed during the study are available from the corresponding author upon reasonable request.

## Supplementary Materials

The following supporting information can be downloaded at https://www.mdpi.com/article/10.3390/v16071144/s1, Table S1: Cats participating in the study (*n* = 40), including signalment, number of additional cats in the household, location of effusion, adverse events and duration (on days of treatment), other diseases before treatment, other unrelated diseases developing during treatment, and additional symptomatic therapy.

## References

[1] Pedersen N.C., Lai M.M.C., Stohlman S.A. (1987). Virologic and immunologic aspects of feline infectious peritonitis virus infection. Coronaviruses.

[2] Murphy B.G., Perron M., Murakami E., Bauer K., Park Y., Eckstrand C., Liepnieks M., Pedersen N.C. (2018). The nucleoside analog GS-441524 strongly inhibits feline infectious peritonitis (FIP) virus in tissue culture and experimental cat infection studies. Vet. Microbiol..

[3] Pedersen N.C., Perron M., Bannasch M., Montgomery E., Murakami E., Liepnieks M., Liu H. (2019). Efficacy and safety of the nucleoside analog GS-441524 for treatment of cats with naturally occurring feline infectious peritonitis. J. Feline Med. Surg..

[4] Addie D.D., Covell-Ritchie J., Jarrett O., Fosbery M. (2020). Rapid resolution of non-effusive feline infectious peritonitis uveitis with an oral adenosine nucleoside analogue and feline interferon omega. Viruses.

[5] Dickinson P.J., Bannasch M., Thomasy S.M., Murthy V.D., Vernau K.M., Liepnieks M., Montgomery E., Knickelbein K.E., Murphy B., Pedersen N.C. (2020). Antiviral treatment using the adenosine nucleoside analogue GS-441524 in cats with clinically diagnosed neurological feline infectious peritonitis. J. Vet. Intern. Med..

[6] Jones S., Novicoff W., Nadeau J., Evans S. (2021). Unlicensed GS-441524-like antiviral therapy can be effective for at-home treatment of feline infectious peritonitis. Animals.

[7] Katayama M., Uemura Y. (2021). Therapeutic effects of Mutian® xraphconn on 141 client-owned cats with feline infectious peritonitis predicted by total bilirubin levels. Vet. Sci..

[8] Krentz D., Zenger K., Alberer M., Felten S., Bergmann M., Dorsch R., Matiasek K., Kolberg L., Hofmann-Lehmann R., Meli M.L. (2021). Curing cats with feline infectious peritonitis with an oral multi-component drug containing GS-441524. Viruses.

[9] Addie D.D., Silveira C., Aston C., Brauckmann P., Covell-Ritchie J., Felstead C., Fosbery M., Gibbins C., Macaulay K., McMurrough J. (2022). Alpha-1 acid glycoprotein reduction differentiated recovery from remission in a small cohort of cats treated for feline infectious peritonitis. Viruses.

[10] Coggins S.J., Norris J.M., Malik R., Govendir M., Hall E.J., Kimble B., Thompson M.F. (2023). Outcomes of treatment of cats with feline infectious peritonitis using parenterally administered remdesivir, with or without transition to orally administered GS-441524. J. Vet. Intern. Med..

[11] Green J., Syme H., Tayler S. (2023). Thirty-two cats with effusive or non-effusive feline infectious peritonitis treated with a combination of remdesivir and GS-441524. J. Vet. Intern. Med..

[12] Taylor S.S., Coggins S., Barker E.N., Gunn-Moore D., Jeevaratnam K., Norris J.M., Hughes D., Stacey E., MacFarlane L., O’Brien C. (2023). Retrospective study and outcome of 307 cats with feline infectious peritonitis treated with legally sourced veterinary compounded preparations of remdesivir and GS-441524 (2020–2022). J. Feline Med. Surg..

[13] Cosaro E., Pires J., Castillo D., Murphy B.G., Reagan K.L. (2023). Efficacy of oral remdesivir compared to GS-441524 for treatment of cats with naturally occurring effusive feline infectious peritonitis: A blinded, non-inferiority study. Viruses.

[14] Yin Y., Li T., Wang C., Liu X., Ouyang H., Ji W., Liu J., Liao X., Li J., Hu C. (2021). A retrospective study of clinical and laboratory features and treatment on cats highly suspected of feline infectious peritonitis in Wuhan, China. Sci. Rep..

[15] Lv J., Bai Y., Wang Y., Yang L., Jin Y., Dong J. (2022). Effect of GS-441524 in combination with the 3C-like protease inhibitor GC376 on the treatment of naturally transmitted feline infectious peritonitis. Front. Vet. Sci..

[16] Krentz D., Zwicklbauer K., Felten S., Bergmann M., Dorsch R., Hofmann-Lehmann R., Meli M.L., Spiri A.M., von Both U., Alberer M. (2022). Clinical follow-up and postmortem findings in a cat that was cured of feline infectious peritonitis with an oral antiviral drug containing GS-441524. Viruses.

[17] Zwicklbauer K., Krentz D., Bergmann M., Felten S., Dorsch R., Fischer A., Hofmann-Lehmann R., Meli M.L., Spiri A.M., Alberer M. (2023). Long-term follow-up of cats in complete remission after treatment of feline infectious peritonitis with oral GS-441524. J. Feline Med. Surg..

[18] Meli M.L., Spiri A.M., Zwicklbauer K., Krentz D., Felten S., Bergmann M., Dorsch R., Matiasek K., Alberer M., Kolberg L. (2022). Fecal Feline Coronavirus RNA Shedding and Spike Gene Mutations in Cats with Feline Infectious Peritonitis Treated with GS-441524. Viruses.

[19] Murphy B.G., Castillo D., Neely N.E., Kol A., Brostoff T., Grant C.K., Reagan K.L. (2024). Serologic, Virologic and Pathologic Features of Cats with Naturally Occurring Feline Infectious Peritonitis Enrolled in Antiviral Clinical Trials. Viruses.

[20] Freeman L., Becvarova I., Cave N., MacKay C., Nguyen P., Rama B., Takashima G., Tiffin R., Van Beukelen P., Yathiraj S. (2011). WSAVA nutritional assessment guidelines. J. Feline Med. Surg..

[21] Thayer V., Gogolski S., Felten S., Hartmann K., Kennedy M., Olah G.A. (2022). 2022 AAFP/Every cat feline infectious peritonitis diagnosis guidelines. J. Feline Med. Surg..

[22] Tasker S., Addie D.D., Egberink H., Hofmann-Lehmann R., Hosie M.J., Truyen U., Belak S., Boucraut-Baralon C., Frymus T., Lloret A. (2023). Feline infectious peritonitis: European advisory board on cat diseases guidelines. Viruses.

[23] Hartmann K. (2005). Feline infectious peritonitis. Vet. Clin. N. Am. Small Anim. Pract..

[24] Dülsner A., Hack R., Krüger C., Pils M., Scherer K., Schmelting B., Schmidt M., Weinert H., Jordan T. Fachinformation aus dem Ausschuss für Tier-Schutzbeauftragte und dem Arbeitskreis 4 in der TVT; Empfehlung zur Blutentnahme bei Versuchstieren, Insbesondere Kleinen Versuchstieren. https://www.gv-solas.de/wp-content/uploads/2017/07/Empfehlung-zur-Blutentnahme_2017-1.pdf.

[25] Hartmann K., Kuffer M. (1998). Karnofsky’s score modified for cats. Eur. J. Med. Res..

[26] Hansen A.E., Schaap M.K., Kjelgaard-Hansen M. (2006). Evaluation of a commercially available human serum amyloid A (SAA) turbidimetric immunoassay for determination of feline SAA concentration. Vet. Res. Commun..

[27] Helfer-Hungerbuehler A.K., Spiri A.M., Meili T., Riond B., Krentz D., Zwicklbauer K., Buchta K., Zuzzi-Krebitz A.-M., Hartmann K., Hofmann-Lehmann R. (2024). Alpha-1-acid glycoprotein quantification via spatial proximity analyte reagent capture luminescence assay: Application as diagnostic and prognostic marker in serum and effusions of cats with feline infectious peritonitis undergoing GS-441524 therapy. Viruses.

[28] Purina Fecal Scoring Chart. https://www.purinainstitute.com/centresquare/nutritional-and-clinical-assessment/purina-fecal-scoring-chart.

[29] Meli M., Kipar A., Muller C., Jenal K., Gonczi E., Borel N., Gunn-Moore D., Chalmers S., Lin F., Reinacher M. (2004). High viral loads despite absence of clinical and pathological findings in cats experimentally infected with feline coronavirus (FCoV) type I and in naturally FCoV-infected cats. J. Feline Med. Surg..

[30] Gut M., Leutenegger C.M., Huder J.B., Pedersen N.C., Lutz H. (1999). One-tube fluorogenic reverse transcription-polymerase chain reaction for the quantitation of feline coronaviruses. J. Virol. Methods.

[31] Lutz H., Hauser B., Horzinek M.C. (1985). Feline infectious peritonitis. Tijdschr. Diergeneeskd..

[32] Osterhaus A.D., Horzinek M.C., Reynolds D.J. (1977). Seroepidemiology of feline infectious peritonitis virus infections using transmissible gastroenteritis virus as antigen. Zentralblatt Veterinärmedizin Reihe B.

[33] Brunner C., Kanellos T., Meli M.L., Sutton D.J., Gisler R., Gomes-Keller M.A., Hofmann-Lehmann R., Lutz H. (2006). Antibody induction after combined application of an adjuvanted recombinant FeLV vaccine and a multivalent modified live virus vaccine with a chlamydial component. Vaccine.

[34] Felten S., Klein-Richers U., Hofmann-Lehmann R., Bergmann M., Unterer S., Leutenegger C.M., Hartmann K. (2020). Correlation of feline coronavirus shedding in feces with coronavirus antibody titer. Pathogens.

[35] Mateos González M., Sierra Gonzalo E., Casado Lopez I., Arnalich Fernández F., Beato Pérez J.L., Monge Monge D., Vargas Núñez J.A., García Fenoll R., Suárez Fernández C., Freire Castro S.J. (2021). The prognostic value of eosinophil recovery in COVID-19: A multicentre, retrospective cohort study on patients hospitalised in spanish hospitals. J. Clin. Med..

[36] Kroegel C., Schreiber J. (2018). Der eosinophile Granulozyt—vom Ursprung bis zur therapeutischen Zielzelle. Z. Pneumol..

[37] Fraissé M., Logre E., Mentec H., Cally R., Plantefève G., Contou D. (2020). Eosinophilia in critically ill COVID-19 patients: A French monocenter retrospective study. Crit. Care.

[38] Kipar A., Meli M.L., Baptiste K.E., Bowker L.J., Lutz H. (2010). Sites of feline coronavirus persistence in healthy cats. J. Gen. Virol..

[39] Mustaffa-Kamal F., Liu H., Pedersen N.C., Sparger E.E. (2019). Characterization of antiviral T cell responses during primary and secondary challenge of laboratory cats with feline infectious peritonitis virus (FIPV). BMC Vet. Res..

[40] Pedersen N.C., Kim Y., Liu H., Galasiti Kankanamalage A.C., Eckstrand C., Groutas W.C., Bannasch M., Meadows J.M., Chang K.O. (2018). Efficacy of a 3C-like protease inhibitor in treating various forms of acquired feline infectious peritonitis. J. Feline Med. Surg..

[41] Slaviero M., Cony F.G., da Silva R.C., De Lorenzo C., de Almeida B.A., Bertolini M., Driemeier D., Pavarini S.P., Sonne L. (2024). Pathological findings and patterns of feline infectious peritonitis in the respiratory tract of cats. J. Comp. Pathol..

[42] Kent A.M., Guan S., Jacque N., Novicoff W., Evans S.J.M. (2024). Unlicensed antiviral products used for the at-home treatment of feline infectious peritonitis contain GS-441524 at significantly different amounts than advertised. J. Am. Vet. Med. Assoc..

