# Supplementary material for: Short Treatment of 42 Days with Oral GS-441524 Results in Equal Efficacy as the Recommended 84-Day Treatment in Cats Suffering from Feline Infectious Peritonitis with Effusion—A Prospective Randomized Controlled Study
Source: Viruses. 2024 Jul 16;16(7):1144. doi: 10.3390/v16071144 (PMC11281457; doi:10.3390/v16071144)
Supplement: Supplementary file 1 [file viruses-16-01144-s001.zip › viruses-3085336-supplementary.pdf]

Table S1. Cats participating in the study ($n = 40$), including signalment, number of additional cats in the household, location of effusion, adverse events and duration (on days of treatment**)**, other diseases before treatment, other unrelated diseases developing during treatment, and additional symptomatic therapy.

Cats	Days of Treatment	Age in Months	Sex	Breed	Additional Cats in the Household (Number)	Location of Effusion	Adverse Events and Duration (on Days of Treatment)	Other Diseases before Treatment	Other Unrelated Diseases Developing during Treatment	Additional Symptomatic Therapy
cat 1	84	16.5	male neutered	BSH	yes (1)	abdominal	diarrhea (40), lymphocytosis (5–42), increased liver enzyme activity (5–28)	chronic gingivo-stomatitis	coughing	fluid therapy [1], mirtazapine [2]
cat 2	42	5.9	male intact	BSH	yes (1)	abdominal	diarrhea (5–28), increased liver enzyme activity (14–28)		pyelectasis	fluid therapy, mirtazapine, maropitant [3], antibiotics [4], probiotics [5]
cat 3	42	6.4	female intact	DSH	yes (1)	abdominal	diarrhea (3–28) increased liver enzyme activity (5–42), eosinophilia (42)			fluid therapy, mirtazapine, antibiotics, probiotics
cat 4	42	14.4	male neutered	Russian blue mix	yes (1)	abdominal	increased liver enzyme activity (1–42), eosinophilia (1–3)			mirtazapine, antibiotics, silymarin [6], probiotics
cat 5	42	98.6	male neutered	DSH	yes (1)	abdominal	diarrhea (7, 23–24), increased liver enzyme activity (42)			mirtazapine, maropitant, antibiotics, probiotics
cat 6	84	10.8	male intact	Somali	no	bicaval (pleural, abdominal)	diarrhea (23–26), lymphocytosis (3–84), increased liver enzyme activity (7–56), eosinophilia (14–84)		calicivirus infection (detected on day 23)	oxygen cage [7], fluid therapy, maropitant, antibiotics, probiotics
cat 7	42	6.8	male intact	DSH	yes (1)	bicaval (pleural, abdominal)	diarrhea (5–7, 14), lymphocytosis (14–42), increased liver enzyme activity (3–42)		intestinal parasite infestation (*Giardia* spp. and *Toxocara* spp., treated with fenbendazole)	probiotics

cat 8	84	11.2	male intact	Maine Coon	yes (1)	abdominal	Heinz bodies 19 % (7) diarrhea (13–28) increased liver enzyme activity (5)	entropion	factor-XI-deficiency, eye infection	mirtazapine, maropitant, antibiotics, probiotics, SAMe [8]
cat 9	84	33.1	male neutered	BSH	yes (1)	abdominal	diarrhea (14) eosinophilia (56)			antibiotics, probiotics
cat 10	42	11.1	male intact	BSH	yes (1)	abdominal	lymphocytosis (5–7), increased liver enzyme activity (5–42), eosinophilia (14–42)			mirtazapine, maropitant, probiotics, silymarin
cat 11	42	90.3	male neutered	DSH	no	abdominal	diarrhea (7), lymphocytosis (5–7)			mirtazapine, maropitant buprenorphine [9], probiotics gabapentin [10]
cat 12	42	42	male neutered	DSH	yes (1)	bicaval (pleural, abdominal)	increased liver enzyme activity (5–7), eosinophilia (28–42)			fluid therapy, mirtazapine, maropitant
cat 13	42	12.4	female intact	Maine Coon	yes (2)	bicaval (pleural, abdominal)	lymphocytosis (5–42), increased liver enzyme activity (7–42)	entropion, conjunctivitis	seizure (day 3)	fluid therapy, mirtazapine, maropitant, antibiotics, probiotics, levetiracetam [11]
cat 14	42	40.5	male neutered	Maine Coon	yes (3)	pleural and trivial pericardial		chronic gingivo-stomatitis cardiography: biatrial dilatation	euthanized (day 31)	oxygen cage, maropitant, mirtazapine, metamizole [12], antibiotics buprenorphine, terbutaline [13], prednisolone [14]
cat 15	84	6	male intact	Maine Coon Mix	yes (1)	pleural	increased liver enzyme activity (56), eosinophilia (14–84)		fever (day 15) with high grade leukocytosis	fluid therapy, oxygen cage, maropitant, mirtazapine, antibiotics, metamizole, probiotics, butorphanol [15]
cat 16	42	12.2	male neutered	Scottish Straight	yes (1)	pleural	increased liver enzyme activity (14–42), eosinophilia (14)		gastroenteritis (day 45) – therapy with fluid maropitant, ondansetron [16]	fluid therapy, mirtazapine, antibiotics, butorphanol

cat 17	84	22.4	male neutered	Scottish Fold	yes (1)	tricaval (pleural, abdominal, trivial pericardial)	diarrhea (12–21), lymphocytosis (84), eosinophilia (14–84)	OCD [17]		fluid therapy, mirtazapine, maropitant, antibiotics, probiotics, buprenorphine, butorphanol
cat 18	42	6.8	female intact	DSH	yes (1)	abdominal	diarrhea (2–21), lymphocytosis (5–28), increased liver enzyme activity (28–42), eosinophilia (28)			fluid therapy, mirtazapine, maropitant, ondansetron, antibiotics, probiotics, buprenorphine, silymarin, SAMe
cat 19	84	14.5	male neutered	Siamese Mix	yes (1)	abdominal	diarrhea (5–14) lymphocytosis (7–56), eosinophilia (28)	pyelectasis	pyelectasis	fluid therapy, maropitant, antibiotics, probiotics, butorphanol, terazosin [18]
cat 20	42	16.8	female neutered	DSH	yes (1)	abdominal	lymphocytosis (28–42)	gingivo-stomatitis	intestinal parasite infestation (*Giardia* spp., treated with fenbendazole)	fluid therapy, mirtazapine, maropitant, ondansetron
cat 21	84	116.3	female intact	Exotic Shorthair	yes (1)	bicaval (pleural, abdominal)		AV-block grade III	sepsis	fluid therapy, mirtazapine, maropitant, ondansetron, antibiotics, buprenorphine, norepinephrine [19], midazolam [20], levetiracetam
cat 22	42	49.5	female intact	DSH	no	abdominal	diarrhea (3–14), lymphocytosis (5–7), increased liver enzyme activity (28–42), eosinophilia (42)			fluid therapy, antibiotics, probiotics
cat 23	84	6.2	male intact	BSH	no	abdominal	diarrhea (5–9), lymphocytosis (7), increased liver enzyme activity (7–14), eosinophilia (28–84)			fluid therapy, maropitant, metamizole, antibiotics, probiotics, silymarin, SAMe
cat 24	84	5.7	male intact	Holy Birman	yes (1)	abdominal	diarrhea (42–84), increased liver enzyme activity (4–84), eosinophilia (42–84)	myocarditis		fluid therapy, mirtazapine, maropitant, ondansetron, antibiotics,

										heart medication [21], clopidogrel [22], eye medication [23]
cat 25	42	6.4	female intact	BSH	no	abdominal	diarrhea (3–14), eosinophilia (18–42)	persistent caninus	IMHA [24]	fluid therapy, mirtazapine, maropitant, ondansetron, antibiotics, prednisolone
cat 26	84	34.9	female intact	DSH	yes (1, cat 37)	abdominal	diarrhea (7–14), increased liver enzyme activity (7)			fluid therapy, mirtazapine, maropitant, antibiotics, probiotics
cat 27	42	11.1	male neutered	DSH	yes (4)	pleural	diarrhea (7–14), increased liver enzyme activity (5), eosinophilia (14–28)			oxygen cage, maropitant butorphanol, antibiotics
cat 28	42	7.5	male neutered	DLH Mix	yes (1)	abdominal	diarrhea (5–21), lymphocytosis (7–28)		mitral valve dysplasia, fleas	fluid therapy mirtazapine, maropitant, antibiotics, probiotics, telmisartan [25]
cat 29	42	18.5	male neutered	DSH	yes (4)	abdominal	diarrhea (3–14) increased liver enzyme activity (7)			fluid therapy, mirtazapine, maropitant, ondansetron, antibiotics, probiotics
cat 30	84	10.7	male neutered	DSH	no	pleural	lymphocytosis (5–42), increased liver enzyme activity (5–28)			fluid therapy, mirtazapine, metamizole, antibiotics, butorphanol
cat 31	84	10.8	male neutered	DSH-BSH Mix	no	abdominal	lymphocytosis (5–84), increased liver enzyme activity (14–84), eosinophilia (42–84)	low grade SAM [26] with suspected mitral valve dysplasia		fluid therapy, mirtazapine, maropitant, ondansetron, antibiotics, buprenorphine
cat 32	84	13	male intact	BSH	no	bicaval (pleural, abdominal)	diarrhea (5–7, 21), lymphocytosis (5–84), increased liver enzyme activity (5–14), eosinophilia (7–84)			fluid therapy, probiotics
cat 33	84	15.4	male neutered	DSH	no	abdominal	diarrhea (28) lymphocytosis (3–84)	pruritus		fluid therapy, esomeprazole [27]

cat 34	84	19.6	male intact	DSH	yes (1)	abdominal	lymphocytosis (7–84), eosinophilia (14–84)			fluid therapy, antibiotics
cat 35	42	9.5	male neutered	Siamese	yes (1)	abdominal	lymphocytosis (14–42), increased liver enzyme activity (28–42), eosinophilia (42)		intestinal parasite infestation (*Giardia* spp., treated with fenbendazole), liver cysts (day 84)	fluid therapy, mirtazapine, maropitant, ondansetron
cat 36	84	5.1	male intact	BSH	yes (1)	bicaval (pleural, abdominal)	diarrhea (3) lymphocytosis (7–84), increased liver enzyme activity (5–7), eosinophilia (56)			fluid therapy, mirtazapine, maropitant, probiotics
cat 37	42	36.1	male neutered	DSH	yes (1, cat 26)	abdominal	diarrhea (3–7, 28–42), lymphocytosis (7–42)		FHV1/ Mycoplasma felis positive	fluid therapy mirtazapine, maropitant, ondansetron, antibiotics, probiotics, buprenorphine
cat 38	84	70.1	male neutered	Ragdoll	no	bicaval (pleural, abdominal)	diarrhea (35) lymphocytosis (7), eosinophilia (84)	myocarditis	eye discharge and fever (day 35), injury (day 62) treated with meloxicam [28] and antibiotics	fluid therapy mirtazapine, maropitant, antibiotics, probiotics, buprenorphine
cat 39	84	104.2	female neutered	OSH	yes (1)	abdominal and trivial pericardial	lymphocytosis (7), increased liver enzyme activity (14)	myocarditis, renal mineralization	pyelectasis (treated with a SUB [29])	mirtazapine, maropitant, ondansetron, antibiotics, clopidogrel, terazosin, fentanyl [30], methadone [31]
cat 40	84	48.1	male neutered	DSH	yes (2)	tricaval (pleural, abdominal, trivial pericardial)	increased liver enzyme activity (5), eosinophilia (42–84)	myocarditis		fluid therapy, mirtazapine, maropitant, atenolol [32]

mg, milligram; kg, kilogram; q24h, every 24 h; IV, intravenous; PO, per os; IM, intramuscular; DLH, Domestic Longhair DSH, Domestic shorthair; BSH, British shorthair; spp., species. [1] fluid therapy with Sterofundin with potassium supplementation at 20 mmol/L for dehydration at an individual dosage calculated by

rehydration and maintenance needs. [2] mirtazapine ointment q24h for appetite stimulation. [3] maropitant 1 mg/kg q24h IV for treatment of gastrointestinal signs, such as vomiting and anorexia. [4] antibiotics (i.e., amoxicillin/clavulanic acid 20 mg/kg q8h IV or PO; marbofloxacin 2 mg/kg q24h IV; pradofloxacin 6 mg/kg q24h PO; doxycycline 8 mg/kg q12h PO). [5] probiotics (Sivomixx®) q12h PO. [6] silymarin 20 mg/kg q12h PO for ten days and then 20 mg/kg q24h PO for treatment of increased liver enzymes. [7] oxygen cage for support in cases of dyspnea in cats with massive pleural effusion. [8] s-adenosylmethionine (SAMe) 20 mg/kg q24h PO. [9] buprenorphine 0.01 mg/kg q8h IV for treatment of pain. [10] gabapentin 20 mg/kg PO for calming before examination [11] levetiracetam 20 mg/kg q8h IV or PO. [12] metamizole 30 mg/kg IV for treatment of fever (body temperature > 40.5 °C) as a single injection. [13] terbutaline 0.01 mg/kg q4h SC. [14] prednisolone 1–2.5 mg/kg q24h PO. [15] butorphanol 0.02–0.03 mg/kg IV or IM for thoracocentesis (sometimes in combination with alfaxalone and midazolam). [16] ondansetron 0.2 mg/kg q8h IV. [17] OCD: osteochondrosis dissecans. [18] terazosin 0.5 mg/cat q24h PO. [19] norepinephrine 0,1–0,5 µg/kg/min IV. [20] midazolam 0.5 mg/kg IM in case of a seizure. [21] heart medication cat 24: *rivaroxaban 0.625 mg/kg q24h PO, furosemide 1.25 mg/kg q12h PO, pimobendan 0.3 mg/kg q12h PO*. [22] clopidogrel 18.75 mg/cat PO. [23] eye medication cat 24 prednisolonacetat, atropine ointment. [24] IMHA: immune-mediated hemolytic anemia. [25] telmisartan 1 mg/kg q24h PO. [26] SAM: systolic anterior motion. [27] esomeprazole 1 mg/kg q12h IV. [28] meloxicam 0.1 mg/kg q24h PO for one day and then 0.05 mg/kg q24h PO. [29] SUB: subcutaneous ureteral bypass, [30] fentanyl 6 µg/kg/h IV for treatment of pain due to the SUB, [31] methadone 0.2 mg/kg q6h IV for treatment of pain due to the SUB, [32] atenolol 1.5 mg/kg q12h PO.
